# Improved Diagnostics by Assessing the Micromorphology of Breast Calcifications via X-Ray Dark-Field Radiography

**DOI:** 10.1038/srep36991

**Published:** 2016-11-14

**Authors:** Kai Scherer, Eva Braig, Sebastian Ehn, Jonathan Schock, Johannes Wolf, Lorenz Birnbacher, Michael Chabior, Julia Herzen, Doris Mayr, Susanne Grandl, Anikó Sztrókay-Gaul, Karin Hellerhoff, Franz Pfeiffer

**Affiliations:** 1 Department of Physics and Institute of Medical Engineering, Technische Universität München, James-Frank-Straße, Garching, Germany; 2 Department of Pathology, Ludwig Maximilian University, Thalkirchner Straße, Munich, Germany; 3 Department of Clinical Radiology, Ludwig Maximilian Universität, Marchioninistraße, Munich, Germany

## Abstract

Breast microcalcifications play an essential role in the detection and evaluation of early breast cancer in clinical diagnostics. However, in digital mammography, microcalcifications are merely graded with respect to their global appearance within the mammogram, while their interior microstructure remains spatially unresolved and therefore not considered in cancer risk stratification. In this article, we exploit the sub-pixel resolution sensitivity of X-ray dark-field contrast for clinical microcalcification assessment. We demonstrate that the micromorphology, rather than chemical composition of microcalcification clusters (as hypothesised by recent literature), determines their absorption and small-angle scattering characteristics. We show that a quantitative classification of the inherent microstructure as ultra-fine, fine, pleomorphic and coarse textured is possible. Insights underlying the micromorphological nature of breast calcifications are verified by comprehensive high-resolution micro-CT measurements. We test the determined microtexture of microcalcifications as an indicator for malignancy and demonstrate its potential to improve breast cancer diagnosis, by providing a non-invasive tool for sub-resolution microcalcification assessment. Our results indicate that dark-field imaging of microcalcifications may enhance the diagnostic validity of current microcalcification analysis and reduce the number of invasive procedures.

In diagnostic mammography breast microcalcifications are the primary evidence in the detection and assessment of early stage breast cancer (carcinoma *in-situ*) and impalpable tumor lesions^1^,^2^. Position, size, shape and appearance of microcalcifications within the breast indicate their possible origin as either benign or cancerous cell processes^3^,^4^,^5^. Morphology and distribution descriptors (BIRADS) help to categorize microcalcifications and stratify the malignancy risk of the corresponding breast tissue, hence determining the need for invasive assessment and follow-up procedures^6^,^7^,^8^. However, a clear assignment as benign or malignant is only possible for a few characteristic microcalcification configurations such as egg, popcorn, skin (benign), fine linear and branching shapes (malignant)^9^. The predominant number of microcalcification types exhibit an unspecific, amorphous, coarse heterogeneous or pleomorphic pattern in the mammogram, which is graded as uncertain and of intermediate concern resulting in a high rate of benign biopsies^10^,^11^,^12^.

Diagnostic potential and validity of current microcalcification analyses is limited by the fact that morphological descriptors are restricted to the global appearance of microcalcification clusters, since their microstructure remains unresolved with clinical mammography systems (resolution of 70–100 μm)^13^. Furthermore, evaluation is susceptible to projection-based superposition effects within the microcalcification cluster^14^. Recent literature however gives strong evidence that spatial information on the interior morphology of microcalcifications could complement and refine the conventional assessment and grading of microcalcifications in clinical examination: Gufler, H. *et al*. reported in *ex-vivo* microCT studies investigating biopsy samples (resolution of 8.4 μm) that benign microcalcifications exhibit a lamellar-trabecular micromorphology while malignant microcalcifications are composed of granulous substructures^15^. Imamura, K. *et al*. used synchrotron radiation imaging (resolution of 6 μm) to demonstrate that pleomorphic microcalcifications consist of a significantly higher ratio of fine, clustered specks than amorphous microcalcifications^16^. Finally, Langen, H. *et al*. revealed calcifications which appeared linear on conventional radiography (12 Lp/mm), thus assigned as high risk calcification, as a superposition of harmless, round microcalcifications by using high-resolution microradiography (2000 Lp/mm)^17^.

Although previous *ex-vivo* case studies showed an improved diagnostic discriminability between malignant and benign microcalcifications by assessment of their 3D-micromorphology for certain indications, the underlying imaging techniques are unsuitable for clinical, *in-vivo* mammography: sufficient resolution of the micromorphology requires detector pixel sizes of an order of magnitude smaller than commonly used in clinical mammography. The implementation of adequate detectors would involve an immensely increased imaging dose, incompatible with the dose limits for clinical mammography.

Here we present an alternative, indirect imaging approach based on a laboratory Talbot-Lau Interferometer (incoherent X-ray source, flat panel detector with pixel size of 127 μm, radiographic imaging mode) meeting the requirements of a clinical implementation in the near future^18^,^19^,^20^. The key factor of the presented method is the simultaneous measurement of absorption (relative decrease in transmission *T*) and dark-field signal (relative decrease in fringe-visibility *V*) with a grating-based phase-stepping approach. These two contrasts allow the determination and comparison of material specific absorption power 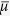 and scattering power 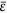 of the investigated specimen^21^,^22^,^23^,^24^,^25^. Malecki, A. *et al*. simulated various microstructure textures composed of randomly distributed spheres and showed that spatial variations of structural parameters far below the detector pixel resolution strongly impact the ratio of absorption 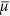 to scatter power 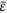
26. Schleede, S. *et al*. utilized this correlation to reveal changes within the microstructure of emphysematous mouse lungs, namely the decay of lung alveoli with disease progression accompanied by a strong decrease in provided scattering power 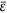, and could successfully discriminate these from healthy lungs^27^.

We adapt this formalism to obtain spatial information on the inherent, sub-resolution structure of microcalcification clusters, by associating 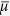 with the overall calcium volume and 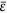 with the calcified interface along the beam in one detector pixel, respectively. The pixel-wise comparison of 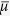 and 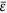 (named “normalized scatter”) then provides a measure of the microscopic surface-to-volume ratio exhibited by the calcium particles enclosed within a particular microcalcification cluster and can be considered as decoupled from the total microcalcification cluster thickness. Consequently, we utilize the mean 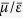-ratio determined over the total calcified area, as a micromorphological classifier, by which differentiation of fine calcium grain configurations (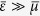) from coarse, compact ones (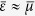) is possible. We test the validity of the proposed classifier by exemplarily correlating the 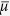 and 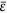 measurements of 15 biopsied microcalcification clusters (covering the full range of micromorphologies), evaluated with grating-based dark-field mammography (at an effective resolution of 85 μm) with separately obtained, highly resolved micro-CT images (at an effective resolution of 6 μm). We further test dark-field mammography as a potential indicator of early tumor malignancy by evaluating 31 biopsied microcalcification clusters with respect to their histopathological findings.

Together with a recent correspondence^28^, our study raises an issue in the conceptual design of a recent publication by Wang, Z. *et al*., in which the 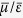-ratio was suggested for microcalcification assessment for the first time, e.g. the differentiation between calcium oxalate dehydrate and calcium hydroxyapatite breast calcifications^29^,^30^. Here the authors relate variations in the 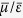-ratio of microcalcifications to their chemical composition/crystalline properties, while overlooking the impact of microstructural properties in scatter-based contrast generation.

## Results

### *In-situ* microcalcification assessment with dark-field mammography

**Figure 1:**
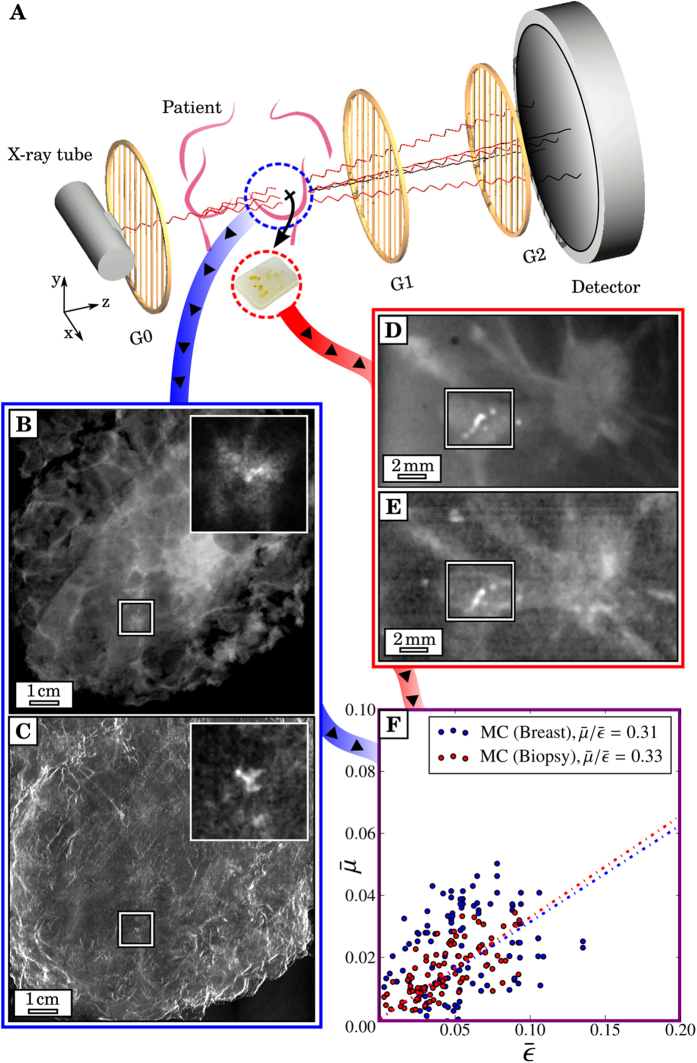
*In-situ* microcalcification assessment with dark-field mammography. (**A**) Sketch of the dark-field mammography setup, consisting of a conventional X-ray tube, a source grating *G*_0_, a phase grating *G*_1_, an analyser grating *G*_2_ and a flat panel detector. Absorption (**B**) and dark-field images (**C**) of a cancerous mastectomy specimen with native microcalcification cluster. The *insets* show magnified images of the microcalcification cluster. Absorption (**D**) and dark-field images (**E**) of the biopsied and paraffin embedded microcalcification cluster. (**F**) Scatter plot comparing absorption 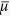 to scattering power 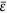 of the native (*blue*) and subsequently biopsied microcalcification cluster (*red*). Mean 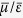-ratios of 0.31 ± 0.02 (Breast) and 0.33 ± 0.01 (Biopsy) are consistent within the error margins, suggesting that microcalcification assessment via dark-field mammography is suitable as an *in-situ* application. Note that the patient graphic was adapted from a breast cancer awareness image (http://freedesignfile.com/upload/2016/02/Breast-cancer-awareness-advertising-posters-pink-styles-vector-02), which is licensed under the Attribution 3.0 Unported license. The license terms (https://creativecommons.org/licenses/by/3.0/) allow adaptation of images, even commercially.

At first, native breast tissue was investigated, to ascertain whether microcalcification assessment with dark-field mammography is clinically applicable. With a lab-based, three grating Talbot-Lau Interferometer (Fig. 1A, Sec. Materials & Methods), absorption (Fig. 1B) and dark-field images (Fig. 1C) of the un-fixated, freshly dissected mastectomy, comprising a distinct microcalcification cluster, were retrieved and stitched from 16 single projections. Subsequently the included cluster was excised, embedded in paraffin and comparative measurements performed, for the purpose of demonstrating that micromorphology classification is achievable independently from the surrounding material composition or thickness. The absorption (Fig. 1D) and dark-field images (Fig. 1E) of the biopsied tissue show the microcalcification cluster in different orientation compared to the mastectomy case.

The measured signals were normalized with respect to the tissue surrounding the microcalcification in order to compensate for absorption and scattering contributions of the underlying tissue in the beam direction as explained in Sec. Materials & Methods. This way, the decrease of beam intensity and fringe visibility caused by only the microcalcification cluster is retrieved. The measured absorption power 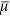 and scattering power 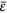 provided by the microcalcification cluster are deduced as the negative logarithm of the normalized transmission (*T*_*c*_) and visibility signals (*V*_*c*_) respectively by


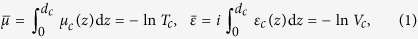


hence equal the integral of linear attenuation coefficient *μ*_*c*_(*z*) and linear diffusion coefficient *ε*_*c*_(*z*) over the spatial mean thickness of the microcalcification cluster *d*_*c*_ respectively, where *i* is an interferometer-specific constant^31^.

For each pixel within the calcified area in the cases of the native breast (blue) and subsequently biopsied microcalcification cluster (red), the values of 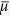 and 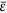 were paired and plotted within a scatter plot (Fig. 1F). Due to a change of cluster orientation with respect to the X-ray beam, accompanied by the excision and embedding process, corresponding projection-based data points differ slightly. However, the overall absorption and scattering power is preserved, since the microscopic surface-to-volume ratio of the microcalcification cluster remains unaltered. The mean 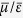-ratios were determined to 0.31(±0.02) in the case of native breast and 0.33(±0.01) in the case of the embedded biopsied microcalcification using a linear least squares fit and were found to be consistent within the error margins. This result demonstrates that the mean 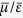-ratio is unaffected by cluster orientation and embedding material/tissue and thus justifies the medical relevance of investigating biopsied microcalcification clusters in this study.

### Dependence of absorption 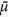 and scattering power 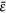 of microcalcification clusters on their inherent micromorphology

**Figure 2:**
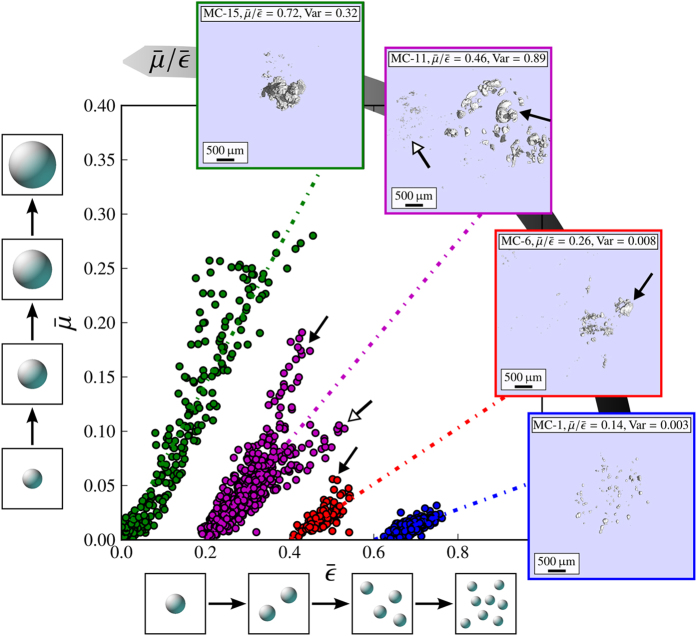
Dependence of absorption and scattering characteristics of microcalcification clusters on their inherent micromorphology. Scatter plots comparing the absorption 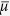 to scattering power 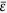 of four microcalcification clusters, representative of the ultra-fine (*blue*), fine (*red*), pleomorphic (*purple*) and coarse (*green*) microtexture classes together with corresponding micro-CT images. For clarity, data points are shifted in 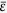-direction by a value of 0.2 each. Discriminative mean 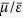-ratios of 0.14, 0.26, 0.46 and 0.72 were found to be strongly different for each of the four microtexture classes. A measure on the degree of regularity in calcium grain sizes is given by the variance of data points. Structural characteristic, i.e. a distinct diversification in grain size in the case of the pleomorphic microtexture, are consistent with the scatter data as indicated by *arrows*.

In order to understand how 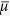 and 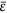 depend on the interior structure of microcalcification clusters, 15 biopsies were additionally investigated with micro-CT measurements (6 μm resolution). In Fig. 2, the data points of four microcalcification clusters with significantly differing microstructures are compared, which are representative of the ultra-fine, fine, pleomorphic and coarse classes. It is apparent that both the slope and pattern of the measured scatter plots vary strongly between the microstructure classes.

The microcalcification with ultra-fine microtexture (blue framed) yields a distinctively uniform micromorphology consisting of very small calcium particles (grain radius of *r* ≈ 75 μm) only. According to expectations, this configuration is strongly scattering and hardly absorbing, resulting in the flattest slope (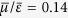) of all investigated data. Furthermore, the spread of data points is small (Var(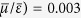), indicating a high degree of regularity in particle size. The microcalcification cluster with fine microstructure (red framed) comprises some additional larger calcium particles (*r* ≈ 175 μm), causing a slight increase in overall absorption power 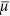, in comparison to the ultra-fine microstructure. Besides, a larger particle correlates well with a few isolated data points (Var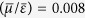), which exceed the average 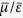-ratio of 0.26 with respect to absorption power 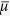. A very pronounced diversification in inherent grain sizes was found in the case of the pleomorphic microcalcification cluster (purple framed). This microstructure comprises a strongly inhomogeneous (Var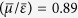), yet separated mix of very small (*r* ≈ 75 μm) and distinctly larger calcium particles (*r* ≈ 275 μm), causing an overall steeper slope of the data points (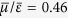), with separation into a strongly scattering/poorly absorbing (small particles for 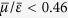) as well as a poorly scattering/strongly absorbing branch (large particles for 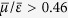), respectively. The steepest slope was found for the microcalcification cluster with coarse texture (green framed). The distinctive feature for this class is a compact micromorphology, comprising very large calcium structures only (*r* ≈ 700 μm) while lacking tiny grains (Var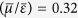). Here the microscopic surface-to-volume ratio exhibited by the microcalcification cluster is minimized, indicated by a strongly increased absorption 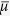 and decreased scattering power 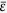, respectively (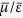 = 0.72). Consequently, by determining the slope and spread (variance) of 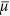 and 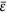 of microcalcification clusters, a measure of the mean particle size and size distribution of calcium grains can be obtained.

To quantitatively investigate the dependence of the 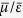-ratio on the microstructure, particle-surface distributions were exemplary deduced: the corresponding micro-CT volumes were thresholded, each microparticle was fitted on a voxel-based segmentation algorithm, its volume and surface interpolated and the total microcalcification surface *σ* accumulated, as explained in Sec. Materials & Methods. The particle-surface distribution then quantifies surface-to-volume characteristics of each cluster, by specifying the relative amount of surface occupied by calcium grains *σ*_*r*_/*σ*, dependent on their grain size *r* (respectively volume).

**Figure 3:**
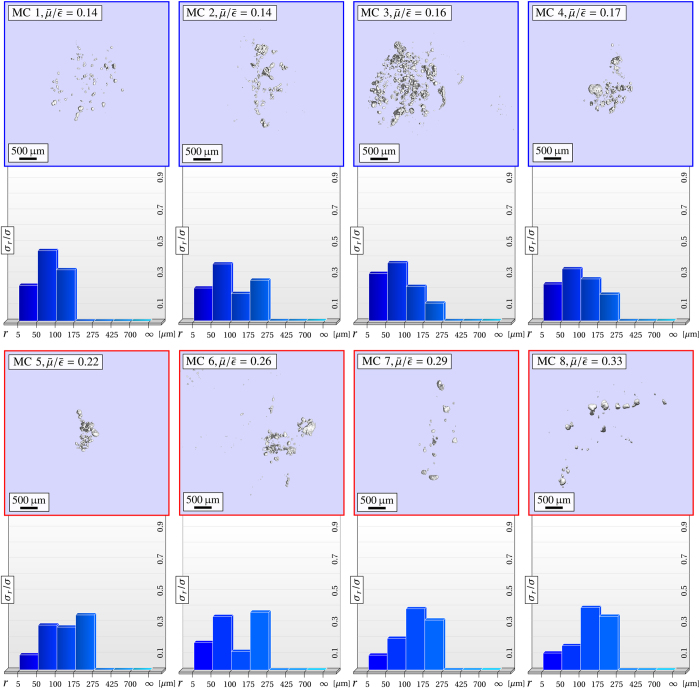
Quantitative classification of microcalcification clusters utilizing the correlation between micromorphology and mean 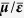-ratio (I). Overview of 8 microcalcification clusters (micro-CT images) sorted with increasing mean 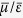-ratio and particle-surface distribution *σ*_*r*_/*σ*, quantifying their inherent microstructure. A consistent relation between the mean 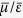-ratio and the micromorphological descriptor *σ*_*r*_/*σ* was found, by which a categorical classification in ultra-fine (*blue*) and fine micromorphologies (*red*) is feasible.

**Figure 4:**
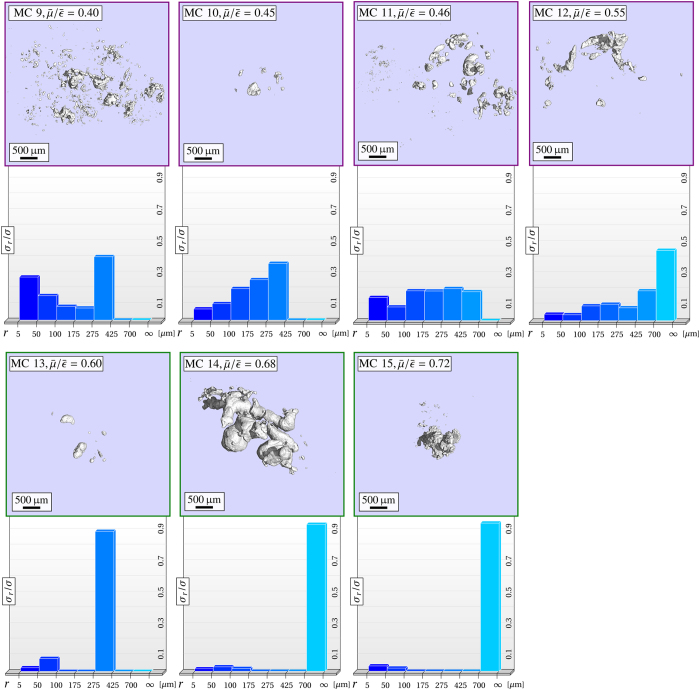
Quantitative classification of microcalcification clusters utilizing the correlation between micromorphology and mean 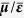-ratio (II). Overview of 7 microcalcification clusters (micro-CT images) sorted with increasing mean 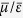-ratio and particle-surface distribution *σ*_*r*_/*σ*, quantifying their inherent microstructure. A consistent relation between the mean 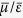-ratio and the micromorphological descriptor *σ*_*r*_/*σ* was found, by which a categorical classification in pleomorphic (*purple*) and coarse micromorphologies (*green*) is feasible. Note that the mean 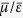-ratio between microcalcification clusters can differ by more then 500%, as for instance when comparing MC 1 against MC 15.

In Figs 3 and 4 we compare the mean 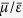-ratios (sorted in increasing order), the interior morphology and particle-surface distributions *σ*_*r*_/*σ* of 15 microcalcification clusters. We found a high correlation between 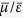 and *σ*_*r*_/*σ* with characteristic values for each of the four microtexture classes, as summarized in Table 1.

Microcalcification clusters, exhibiting a mean 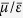-ratio below 0.33 (MC1-8), were found to be exclusively composed of calcium grains smaller than 275 μm in radius. Within this class, microcalcifications meeting the requirement of 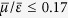 could be further differentiated as ultra-fine (MC1-4) where more then 70% of the overall cluster surface is occupied by ultra small grains (*r* ≤ 175 μm). Microcalcifications with fine texture (MC5-8) yielded a 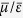-ratio between 0.22 and 0.33 and comprised more of slightly larger particles (175 ≤ *r* ≤ 275 μm) which contribute to more then 30% of the overall surface. The smallest 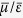-ratio of 0.14 was obtained for an ultra-fine microcalcification cluster (MC1), with no grains bigger than 175 μm in radius.

Four 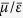-ratios between 0.4 and 0.55 were measured for microcalcification clusters with strongly pleomorphic microstructure (MC9-12). Intermediate 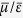-ratios can be attributed to a broad spectrum of included grain sizes and therefore averaging of scattering and absorption contributions related to small and larger particles, respectively. The pleomorphic nature of this class is well indicated by the circumstance that large calcium grains (*r* ≥ 275 μm) occupy at least 0.25% but not more then 0.75% of the overall surface. Consistently, also within this group of microcalcifications a progression towards the inclusion of larger particles (*r* ≥ 425 μm) with increasing 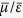-ratio (MC11-12) was identifiable.

Coarse microtextures were observed for microcalcification clusters with 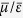-ratios exceeding a threshold of 0.6 (MC13-15). A characteristic for this configuration is a small number of very large calcium structures dominating the overall microtexture appearance, i.e. accounting for more then 85% of the overall surface, while small scatter-determining grains are negligible with respect to the occupied surface fraction. In accordance, the highest 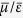-ratio of 0.72 was observed for a structure (MC15) consisting of a single, compact calcification only.

### Microtexture of microcalcifications as a potential indicator for early tissue malignancy

**Figure 5:**
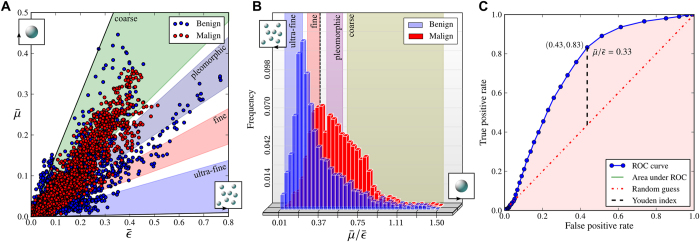
Microtexture of microcalcifications as a potential indicator for early tissue malignancy. (**A**) Scatter plot comparing the absorption 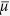 to scattering power 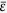 of 11 microcalcifications associated histopathologically with a ductal carcinoma *in-situ (red*) and 20 microcalcifications associated with a benign finding (*blue*). (**B**) Histogram comparing the distribution of all 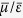-ratios found for benign and malignant microcalcifications obtained from (**A**). Benign microcalcifications yield 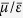-ratios associated with ultra-fine and fine micromorphologies, while malignant microcalcifications prevail within ratios determined for pleomorphic and coarse microstructures. (**C**) Receiver operating characteristic curve estimating the performance of microtexture analysis as a potential indicator for early tissue malignancy, using a single 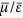-cut-off value. An optimal criterion (Youden index) for the discrimination of begin from malignant microcalcification was found for 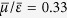.

In a very first approach, we tested the determined microtexture (ultra-fine, fine, pleomorphic or coarse) of microcalcifications as such as an indicator for tissue malignancy in early stages. This was done by comparing 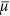 versus 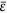 in a scatter plot for 11 microcalcifications associated histopathologically with a ductal carcinoma *in-situ* and 20 microcalcifications associated with a benign finding (Fig. 5A). The overall amount of investigated material corresponds to a microcalcified surface area of approximately 200 mm^2^.

It is noticeable that benign microcalcifications dominate the region of 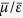-ratios determined for ultra-fine and fine micromorphologies, while the malignant microcalcifications prevail within ratios determined for pleomorphic and coarse microstructures. The frequency distributions of 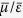-ratios (range of 0.01 to 1.5) obtained for the corresponding benign and malignant microcalcifications (Fig. 5B), ascertain this impression. By relative comparison of the histograms, we found that only 12% and 37% of microcalcifications associated with ultra-fine and fine microstructure are of malignant nature while it is 68% and 69% in case of polymorphic and coarse microtextures, respectively.

In order to utilize this discriminability for diagnostics and estimate the performance of dark-field mammography as a predictor of malignancy, utilizing a single 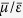-cut-off value, a receiver operating characteristic was obtained^32^. The receiver operating characteristic curve (Fig. 5C) compares sensitivity (true positive rate) against 1-specificity (false positive rate) when classifying microcalcifications as malignant if exceeding various 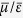-cut-off values. An optimal discrimination criterion (Youden index) was found for 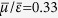 which surprisingly corresponds to the crossover of fine granular to pleomorphic microstructure. With a probability of 83%, a malignant microcalcification will be correctly assigned as such, while 43% of benign microcalcifications are falsely classified. We found that a malignant microcalcification may be graded with higher suspicion than a benign microcalcification utilizing its microtexture a probability of 73% (area under the receiver operating characteristic curve), which is considered a fair test quality^33^.

## Discussion

In summary we have presented a novel and non-invasive method using X-ray dark-field mammography for breast microcalcification assessment that overcomes the major shortcomings of current microcalcification evaluation with conventional mammography: first, microscopic resolution is provided through comparison of the global absorption to scattering power of microcalcification clusters, hence it is unrestricted by the resolution limit of the detector and therefore clinically compatible. Second, microcalcification analysis is quantitative and thus independent of the examining radiologist. A categorical differentiation of microcalcification clusters as ultra-fine, fine, pleomorphic and coarse microtextured was achieved by determining characteristic 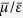-ratios and revealing the particle-surface distribution *σ*_*r*_/*σ* as a suitable micromorphological descriptor.

In a preliminary study investigating 31 biopsy samples the microtexture of microcalcifications was reviewed as a potential indicator for early tumor malignancy, considering that 85–95% cases of ductal carcinoma *in-situ* are detected due to their appearance in screening^1^. A fairly good correlation of ductal carcinoma *in-situ* with pleomorphic and coarse microstructures was reported in our study. Furthermore, we found that the crossover from fine to pleomorphic microstructure could be a potential indicator towards a higher probability of malignancy. Although literature currently does not provide a consistent framework for the validation and interpretation of our results (due to a very limited number of micro-CT studies available), we believe that with ongoing research microtexture analysis utilizing a single cut-off value could provide a differentiated reader-independent tool for risk stratification. Future applications in ductal carcinoma *in-situ* (DCIS) evaluation may include the determination of subtypes or evaluation of developing invasive breast cancer, currently impossible with conventional microcalcification evaluation, with the ultimate goal of addressing over-treatment of DCIS^34^,^35^.

In addition X-ray dark-field mammography could be of high interest for imaging of very small or low density calcifications. We found that ultra-fine microstructures yielded an approximately 7 times higher signal in the dark-field in comparison to the absorption channel (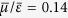). This significant increase in dark-field and reduction of absorption contrast with decreasing calcium grain size, could enable the detection of incipiently growing microcalcifications in early tumor stages or dense breast tissue, which are currently overlooked in conventional mammography^36^,^37^.

Finally, our results strongly indicate that the micromorphology of microcalcifications is the most prevailing factor in the determination of the 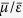-ratio of microcalcifications, which is in accordance with theoretical considerations on the generation of the dark-field imaging contrast^21^,^26^,^38^. First, we observed a change in the 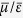-ratio of more then 500% from ultra-fine to coarse microtexture (cf. Fig. 3/4 MC 1 vs. MC 15). Second, minor structural deviations within one microtexture class, for example an increase in maximum grain size, were found to be consistent with a corresponding change in the mean 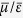-ratio (cf. Fig. 3 MC 1 vs. MC 3). However, based on the resolution of the micro-CT measurements, we can not exclude the influence of an even finer microstructure in the range of the *G*_1_ grating period, independent of the surface-to-volume ratio. Still, the evaluation of this ratio shows a clear correlation with the 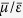-ratio for all calcifications investigated in this study.

**Figure 6:**
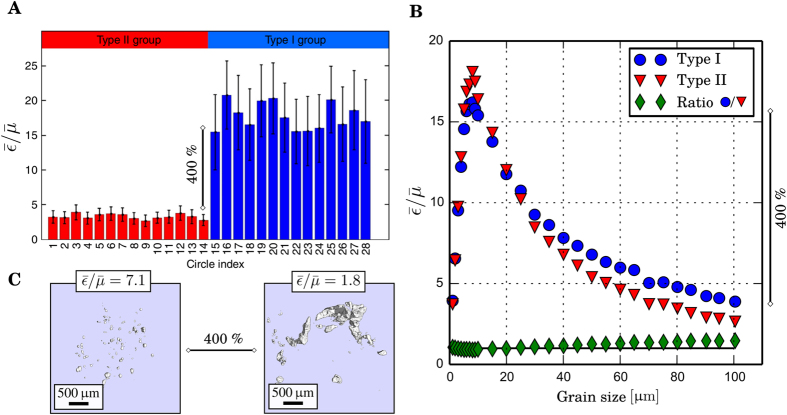
Chemically-based differentiation of microcalcification via dark-field mammography? (**A**) Phantom study investigating potentially arbitrary powders, proposes a chemically/crystallinity-sensitive differentiation of Type I and II microcalcifications, contingent on variations in the exhibited 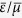-ratios of up to 400%. Supposition and figure adapted from Wang, Z. *et al*.^29^. (**B**) Simulations - based on Malecki, A *et al*.^26^ - showing the 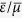-ratio of an assembly of type I and II grains with varying grain size and fixed overall volume. The parameters of the simulated grating interferometer were chosen to be comparable with the experimental setup used in Wang, Z. *et al*.^29^. While the grain size is strongly impacting the 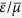-ratio, no significant chemical dependence was found, as illustrated by the *green diamonds* being nearly unity. (**C**) Experimental verification that the reported variations of 400%, can be solely explained by the microstructural properties of calcifications, which renders a purely chemically-based discrimination unlikely. Note that for reasons of consistency the 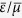
- rather than the 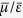-ratio is displayed.

These insights dispute the main conclusion of a recent publication by Wang, Z. *et al*.^29^, namely the differentiation of calcium oxalate dehydrate (Type I) from calcium hydroxyapatite (Type II) breast calcification by measuring the 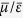-ratio^28^,^29^,^30^. In their study, the authors overlook the impact of microstructural properties on scatter-contrast generation and instead solely relate the 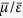-ratio of breast calcification to their chemical composition, on which the proposed diagnostic test is based on. Within their phantom study, they interpret discrepancies in the 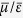-ratio of about 400%, which are very similar to the values presented here, to crystalline properties of type I and type II calcifications, while neglecting that the investigated powders may comprise arbitrary grain sizes (Fig. 6A). Further, our simulation (based on ref. 26) on dark-field imaging of type I and type II calcifications at a grating interferometer comparable to the experimental setup used by Wang, Z. *et al*.^29^ do not predict any significant differences in the exhibited 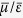-ratios (Fig. 6B), which is in accordance with literature^39^. Hence, our results indicate both a theoretical (Fig. 6B) basis that contradicts the sensitivity for chemical typing of calcifications as well as an experimental basis (Fig. 6C), that the reported variations of 400% could be perfectly correlated with the microstructural constitution of the investigated microcalcification cluster. We are therefore convinced that a purely chemically-based discrimination of calcifications via dark-field radiography is unjustified.

We suggest that the assessment of microcalcifications using dark-field mammography may be suitable as a future *in-situ* application, since we could assign a cluster within native breast tissue as fine textured before undergoing excision and subsequently validating our prediction. In this proof-of-principle study (investigating mostly biopsy samples), long exposure scans (9 phase-steps with 9 seconds exposure time each) were used, resulting in a total mean glandular dose of 72 mGy per projection when measuring the cancerous mastectomy. While this value exceeds clinical dose levels (2.5 mGy) by far, please note that first dose-compatible phase-contrast mammography measurements have been reported recently^40^. Nevertheless, future studies have to investigate to what extend microcalcification assessment can be translated towards low-dose dark-field radiography, e.g. whether a precise differentiation of various micromorphologies is still possible in the case of low contrast-to-noise ratios.

Together with other recently demonstrated advances of dark-field mammography in breast microcalcification imaging such as overall increased contrast-to-noise ratio and enhanced delineation, we believe that microtexture analysis will push grating-based mammography towards clinical application in the near future^41^,^42^. Especially in the large group of uncertainly graded microcalcifications, microtexture analysis yields the potential to refine conventional, highly subjective BIRADS classification and help to improve cancer risk stratification with the final goal of avoiding unnecessary and cost-intensive procedures.

## Materials and Methods

### Ethical approval

The study was conducted in accordance with the Declaration of Helsinki and was approved by the local ethics committee (Ethikkommission of the Ludwig-Maximilian-University, Munich, project number 240-10, date of permission 26/08/2010, amendment 30/05/2012). Inclusion criteria were indication for surgical removal of a benign or malignant breast tumor after previous core biopsy. Participants gave written informed consent before participation after adequate explanation of the study protocol. Indication for breast surgery followed recommendation of the interdisciplinary tumor board of the University of Munich breast center.

### Talbot-Lau interferometer

X-ray dark-field microcalcification assessment was conducted with a compact laboratory setup using a three-grating Talbot-Lau interferometer (effective pixel-size of 85 × 85 μm^2^). The source is a Nonius FR 591 rotating anode tube with molybdenum target (no beam filtration) and focal spot of 0.3 × 0.3 mm^2^, operated at 40 kVp and 70 mA. An indirect X-ray conversion Varian Paxscan 2550*D* flat panel with DRZ-Plus Gadox screen (Gd_2_O_2_S:Tb, 208 μm phosphor layer) coupled with a silicon TFT and 127 × 127 μm^2^-pixel size was implemented as a detector. The beam-splitter grating *G*_1_ is a *π*/2-shifting binary phase-grating with a design energy of 27 keV. The interferometer is built in an asymmetric geometry with periods of 10 μm, 3.24 μm and 4.8 μm for the *G*_0_, *G*_1_ and *G*_2_, respectively. The setup length is 1570 mm, with inter-grating distances of 1060 mm between *G*_0_ and *G*_1_ and 510 mm between *G*_1_ and *G*_2_, corresponding to the third Talbot order at the design energy. The samples were positioned 2.6 cm upstream of *G*_1_. Corresponding source-specimen and specimen-detector distances were determined to 1134 mm and 556 mm, respectively. Simulations on the (sub-resolution) dark-field sensitivity of the utilized setup in dependence of microcalcification grain size are shown in Fig. 6B.

### Pool of specimens

The freshly dissected, un-fixated breast specimen was obtained by mastectomy from a 63 year old patient. Histopathological work-up confirmed an invasive carcinoma. The specimen was measured in cradiocaudal orientation and was compressed to a medically reasonable thickness of 4.5 cm. The pool of 31 biopsies consisted of 11 microcalcifications hist-pathologically associated with *in-situ* carcinomas obtained from 10 patients and 20 microcalcifications hist-pathologically associated with benign finding from 13 patients. In order to avoid small-angle scatter from surface roughness, paraffin blocks were flattened before dark-field measurements.

### Data normalization

For the purpose of investigating microcalcifications independently from the underlying tissue in the beam direction, measured signals (*T, V*) were normalized with respect to the surrounding tissue (*T*_*s*_, *V*_*s*_). Microcalcifications were discriminated from surrounding tissue, by thresholding and subsequent masking of the calcified areas within the dark-field image. On the supposition that the microcalcification cluster is distinctively thinner than the specimen (*d*_*c*_ ≪ *d*), the decrease of beam intensity (*T*_*c*_) and fringe visibility (*V*_*c*_) caused only by the microcalcification cluster can be retrieved by


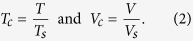


### Micro-CT measurements

High resolution 3D-images were obtained with a micro-CT vtomex scanner (Phoenix X-ray, GE) tuned at 100 μA and 60 kVp. Each paraffin block was measured at 1000 angles with 1 second exposure time per projection. An effective resolution of 6 μm was achieved. 3D-volumes were reconstructed using the software VGStudio Max.

### Voxel-based segmentation algorithm

Micro-CT volume data was segmented by thresholding into two phases - microcalcification and enclosing paraffin - followed by a labelling process, which identifies clusters of interrelated voxels. This was achieved by calculating sub-volumes of voxels, on the condition of at least sharing one mutual surface and considering sub-volumes greater than 5 voxels only. Afterwards, the volume and surface of each particle were interpolated and the particle radius calculated from a sphere of equivalent volume.

### Simulation parameters

The simulated X-ray grating interferometer was based on the wave-optical numerical framework of Malecki, A *et al*.^26^. The X-ray source was considered to emit monochromatic photons at the energy of *E*_*ph*_ = 28 keV in coherent plane waves. The parameters of the grating interferometer were chosen to be comparable with the experimental setup used in Wang, Z. *et al*.^29^ with grating periods of 3.5 μm and 1.75 μm for *G*_1_ and *G*_2_, respectively. It was implemented as a *π*-shifting setup operated in the 5^*th*^ fractional Talbot distance with *d*_*T*_ = 17.29 cm. The wavefront was simulated with a resolution of 87.5 nm. The effective detector pixel size was chosen to be *p*_*eff*_ = 250 μm.

### Dose

The applied radiation dose was calculated by measuring the incident air kerma (2.1 mGy/s) with a Dosimax plus/RQX-detector system. The mean glandular dose was calculated to 0.89 mGy/s by using a Monte-Carlo based conversion factor of 0.422, determined by the half-value layer (Al) of 0.8 mm and a sample thickness of 4.5 cm^43^,^44^. A total mean glandular dose of 72 mSv (9 phase steps with 9 seconds exposure time each) per projection was found.

## Additional Information

**How to cite this article**: Scherer, K. *et al*. Improved Diagnostics by Assessing the Micromorphology of Breast Calcifications via X-Ray Dark-Field Radiography. *Sci. Rep.* **6**, 36991; doi: 10.1038/srep36991 (2016).

**Publisher’s note**: Springer Nature remains neutral with regard to jurisdictional claims in published maps and institutional affiliations.

## Figures and Tables

**Table 1:** Quantitative classification of microcalcification clusters.

Microcalcification	Microtexture Class	*σ*_*r*_/*σ*	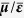
MC1-4	Ultra-Fine (*r*_max_ ≤ 275 μm)	[*σ*_*r*≤175μm_/*σ*] ≥ 0.7	≤0.17
MC5-8	Fine (*r*_max_ ≤ 275 μm)	[*σ*_*r*≤175μm_/*σ*] ≤ 0.7	0.22–0.33
MC9-12	Pleomorphic	0.25 ≤ [*σ*_*r*≥275μm_/*σ*] ≤ 0.75	0.40–0.55
MC13-15	Coarse	[*σ*_*r*≥275μm_/*σ*] ≥ 0.85	≥0.6

Ensuing from Fig. 3/4 microcalcification clusters can be quantitatively differentiated as ultra-fine, fine, pleomorphic and coarse textured, utilizing the correlation between mean 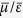-ratio and particle-surface distribution *σ*_*r*_/*σ* (micromorphological descriptor).
